# Circulating testosterone levels and health outcomes in chronic obstructive pulmonary disease: results from ECLIPSE and ERICA

**DOI:** 10.1136/bmjresp-2022-001601

**Published:** 2023-06-14

**Authors:** Holly Pavey, Michael I Polkey, Charlotte E Bolton, Joseph Cheriyan, Carmel M McEniery, Ian Wilkinson, Divya Mohan, Richard Casaburi, Bruce E Miller, Ruth Tal-Singer, Marie Fisk

**Affiliations:** 1 Division of Experimental Medicine and Immunotherapeutics, Department of Medicine, University of Cambridge, Cambridge, UK; 2 Department of Respiratory Medicine, Royal Brompton Hospital, London, UK; 3 Centre for Respiratory Research, Translational Medical Sciences, School of Medicine, University of Nottingham, Nottingham, UK; 4 NIHR Nottingham Biomedical Research Centre, Nottingham, UK; 5 Cambridge University Hospitals NHS Foundation Trust, Cambridge, UK; 6 Former employee of GSK, Collegeville, Pennsylvania, USA; 7 Rehabilitation Clinical Trials Center, Lundquist Institute for Biomedical Innovation at Harbor-UCLA Medical Center, Torrance, California, USA

**Keywords:** COPD epidemiology, COPD Exacerbations, Clinical Epidemiology

## Abstract

The relationship of circulating testosterone levels with health outcomes in people with chronic obstructive pulmonary disease (COPD) is unknown.

**Aim:**

To determine whether serum testosterone levels predict hospitalised acute exacerbations of COPD (H-AECOPD), cardiovascular disease outcome, and mortality in people with COPD.

**Methods:**

Separate analyses were carried out on two observational, multicentre COPD cohorts, Evaluation of COPD Longitudinally to Identify Predictive Surrogate End-points (ECLIPSE) and Evaluation of the Role of Inflammation in Chronic Airways Disease (ERICA), both of which had serum testosterone measured using a validated liquid chromatography assay at the same laboratory. Data from 1296 male participants in ECLIPSE and 386 male, 239 female participants in ERICA were analysed. All analyses were sex-specific. Multivariate logistic regression was used to determine associations with H-AECOPD during follow-up (3 years ECLIPSE, 4.5 years ERICA), a composite endpoint of cardiovascular hospitalisation and cardiovascular death, and all-cause mortality.

**Results:**

Mean (SD) testosterone levels were consistent across cohorts; 459 (197) and 455 (200) ng/dL for males in ECLIPSE and ERICA, respectively, and in ERICA females: 28 (56) ng/dL. Testosterone was not associated with H-AECOPD (ECLIPSE: OR: 0.76, p=0.329, ERICA males: OR (95% CI): 1.06 (0.73 to 1.56), p=0.779, ERICA females: OR: 0.77 (0.52 to 1.12), p=0.178) or cardiovascular hospitalisation and death. Testosterone was associated with all-cause mortality in Global Initiative for Obstructive Lung Disease (GOLD) stage 2 male patients only, in ECLIPSE (OR: 0.25, p=0.007) and ERICA (OR: (95% CI): 0.56 (0.32 to 0.95), p=0.030).

**Conclusions:**

Testosterone levels do not relate to H-AECOPD or cardiovascular outcome in COPD, but are associated with all-cause mortality in GOLD stage 2 COPD male patients, although the clinical significance of this finding is uncertain.

**WHAT IS ALREADY KNOWN ON THIS TOPIC**

- Individuals with chronic obstructive pulmonary disease (COPD) often have manifestations of accelerated ageing and endogenous circulating testosterone levels decline with age. Lower levels of testosterone have been associated with lower muscle mass and strength and increased cardiovascular risk in some population studies. Small studies have suggested that individuals with COPD have low testosterone levels in comparison to controls. It is helpful to evaluate endogenous levels of circulating testosterone’s association with health outcomes in COPD.

**WHAT THIS STUDY ADDS**

- Over a follow-up period of 3–4 years, testosterone levels did not relate to hospitalised COPD exacerbations, cardiovascular hospitalisation or cardiovascular death, but were associated with all-cause mortality in Global Initiative for Obstructive Lung Disease (GOLD) stage 2 male patients with COPD in both cohorts.

**HOW THIS STUDY MIGHT AFFECT RESEARCH, PRACTICE OR POLICY**

- Circulating testosterone levels are associated with all-cause mortality in male patients with COPD GOLD stage 2. Although levels of circulating testosterone are an attractive biomarker, our results do not support the prognostic utility of testosterone testing specifically in patients with COPD. The clinical relevance of testosterone’s association with all-cause mortality in GOLD stage 2 male patients with COPD only may reflect its association with mortality observed in elderly men in the general population, an association which is not observed in males with severe to very severe COPD, or females with COPD.

## Introduction

Circulating testosterone levels naturally decline with age. Circulating testosterone has an independent, inverse association with all-cause mortality in elderly men and low circulating testosterone is associated with lower muscle mass and strength in male population studies.^1 2^ Randomised controlled trials in elderly hypogondal men have suggested improvements in muscle mass and handgrip strength, but not in walking distance with supplementary testosterone therapy.^3–6^

Individuals with chronic obstructive pulmonary disease (COPD) often have manifestations of accelerated ageing including skeletal muscle dysfunction, encompassing reduced muscle mass and strength. Multiple underlying factors are proposed to contribute to skeletal muscle dysfunction in COPD, including physical inactivity, airflow limitation, oxidative stress, inflammation and cachexia.^7 8^ Low circulating testosterone levels are also considered to be a potential and modifiable risk factor of skeletal muscle dysfunction in COPD. Compared with men without COPD, men with COPD tend to have lower levels of testosterone.^9^ Many of the studies which have evaluated testosterone supplementation in COPD have been carried out in specific subgroups of men with COPD, including those with either defined androgen deficiency or documented cachexia, and have demonstrated improvements in muscle mass, body mass index (BMI), health-related quality of life scores and peak work rate in incremental exercise tests, but no improvements in 6 min walk distance (6MWD) or hand grip strength.^9–11^

Besides an association with muscle mass and strength, testosterone treatment has wide ranging effects, from mood enhancement to erythropoiesis,^12 13^ and higher levels of free testosterone as well as testosterone treatment have been shown to reduce risk of asthma in men and women and asthma-related hospitalisations in women.^14^ The association between testosterone and cardiovascular risk is uncertain, with contrasting results from population studies reporting either beneficial or harmful associations.^15–18^ In people with COPD, the clinical significance of circulating testosterone levels in relation to health outcomes such as acute exacerbations resulting in hospitalisation, cardiovascular risk, or all-cause and cause-specific mortality are unknown. Moreover, although levels of circulating testosterone have a crucial role in women too, the importance of testosterone in people with COPD has thus far only been examined in men and studies of testosterone levels in women are generally scarce.

The ECLIPSE (Evaluation of COPD Longitudinally to Identify Predictive Surrogate End-points) and ERICA (Evaluation of the Role of Inflammation in Chronic Airways Disease) studies are uniquely positioned to examine testosterone levels in COPD, as large, prospective, longitudinal observational studies containing well-characterised patients with COPD, which included muscle measures as well as health outcomes data.^19 20^

The primary objective of this analysis was to determine whether levels of circulating total testosterone (TT) were independently related to hospitalisation for acute exacerbations of COPD (H-AECOPD) and mortality. In ERICA, we also looked at length of stay (LOS) for H-AECOPD and cardiovascular outcomes. We also sought to explore cross-sectional relationships of circulating testosterone levels with measures of functional capacity, skeletal muscle strength and surrogate markers of cardiovascular risk.

## Methods

### Study design and study population

We carried out analyses on two separate large, observational, multicentre COPD cohorts, ECLIPSE and ERICA. Methods for both studies have been described in detail previously.^19 20^ Briefly, ECLIPSE^20 21^ was a non-interventional, observational study conducted at 46 centres, in 12 countries, that was designed to determine underlying mechanisms of COPD disease progression and identify biomarkers that may be used as surrogate endpoints of disease progression. All participants were followed up for 3 years from study enrolment and baseline measurements. COPD participants were aged 40–75 years, with baseline postbronchodilator forced expiratory lung volume in 1 s (FEV_1_) divided by forced vital capacity (FVC)<70% and FEV_1_<80% predicted (Global Initiative for Obstructive Lung Disease (GOLD) stage ≥2). The original work was done to support a clinical development project where testosterone assessment in males was planned as a safety measure, therefore, we have not been able to include females from ECLIPSE in these analyses.

ERICA is a multicentre UK observational study that evaluated the role of inflammation in COPD-associated comorbidities (namely, cardiovascular and musculoskeletal).^20^ Participants were studied at baseline and linked for future UK National Health Services (NHS) electronic healthcare records (ie, Hospital Episode Statistics (HES)) and Office of National Statistics (ONS) record of mortality. Participants were aged ≥40 years, with physician-diagnosed COPD and postbronchodilator FEV_1_/FVC<70% and FEV_1_<80% predicted. The analyses presented here from the ERICA study included both COPD men and women. Patients were enrolled in the study in 2011–2014; and collection of HES and ONS follow-up data were censored up to August 2017. The median (IQR) duration of follow-up was 4.5 (4.1–5.0) years.

The ECLIPSE study (registered on ClinicalTrials.gov with identifier NCT00292552 10; GlaxoSmithKline study code SCO104960).

Given the differences in study cohorts, data from each study were analysed individually.

### Patient and public involvement

Patients or the public were not involved in the design, or conduct, or reporting, or dissemination plans of our research.

### Outcomes of interest

#### Hospitalised acute exacerbations of COPD

In ECLIPSE, H-AECOPD, which were defined as COPD exacerbations requiring hospital admission, and were identified by investigators at study scheduled follow-up visits (3 months, 6 months and then every 6 months for a maximum of 3 years). H-AECOPD were identified based on case report forms, participant’s recall of exacerbations or available medical records for exacerbation events, with additional monthly phone calls supplementing information. Distinct exacerbations were defined as exacerbations that started a minimum of 7 days after the end of a prior episode (recovery being defined by the patient).^21 22^

In the ERICA cohort, H-AECOPD were identified from HES data (online supplemental table S1). Admission and discharge dates were used to determine hospital LOS (ie, number of days) evaluated for the first H-AECOPD episode.

10.1136/bmjresp-2022-001601.supp1 Supplementary data

For evaluation of rate of exacerbations in ERICA, an exacerbation was defined as a separate AECOPD event if ≥14 days apart based on admission and discharge dates available from HES data, as per previously published analysis.^23^ The rate of exacerbations was calculated as the number of exacerbations in the follow-up period or until the date of death, whichever came first, divided by the follow-up period. Rates were expressed as rate of H-AECOPD per year. In the ECLIPSE cohort.

### Cardiovascular composite of hospitalisation and CV-related mortality

In the ERICA cohort, hospital admissions related to cardiovascular disease (CVD) were collated using HES data during the follow-up period of the study. CVD was defined based on classifications used by the Emerging Risk Factors Collaboration^24^ (see online supplemental table S2). Cardiovascular-related mortality in the ERICA cohort was based on adjudication of the ONS death certification and has previously been published.^25^ Given the low number of cardiovascular-related deaths in the study (n=15 (2.4%)), a composite endpoint of cardiovascular-related hospitalisation and mortality was used. Cardiovascular hospitalisation or mortality data were not available from the ECLIPSE cohort.

### Mortality

All-cause mortality data were recorded for both studies and included date of death. Cause-specific mortality for respiratory or cardiovascular cause was recorded in ERICA using the ONS death data provided by NHS Digital and adjudicated by cardiovascular and pulmonary physicians.

### Serum testosterone

For both cohorts, TT levels were analysed from serum samples using the same validated liquid chromatography tandem mass spectrometry method at the same laboratory.^26^ Testosterone was measured by liquid chromatography/tandem mass spectrometry. The lower limit of quantification (LLOQ) is 2 ng/dL. The reference range is 265–973 n/dL for men. The between run precision is <10%.

### Other variables

Both ECLIPSE and ERICA studies included key baseline measurements of BMI, 6 min walk test (6MWT) and spirometry. ERICA additionally carried out fat free mass (FFM) measurement by bioelectrical impedance analysis, the quadriceps maximal volitional test (QMVC), and the short physical performance battery (SPPB), as well as non-invasive surrogate markers of cardiovascular risk assessments, namely aortic pulse wave velocity (aPWV) and carotid intima–media thickness (cIMT). Methods for ERICA variables of interest have previously been published.^20^ ECLIPSE additionally had data on full lung function measures.

## Statistical methods

Data from male participants in ECLIPSE and male and female participants in ERICA were analysed separately, of whom all had TT, age, BMI, FEV_1_ and smoking status measured. Baseline characteristics were summarised using mean±SD (or median IQR if appropriate). Spearman’s rank correlation was used to assess associations with age, BMI, FEV1, 6MWT distance, body mass index, airflow Obstruction, Dyspnoea and Exercise (BODE) index and modified Medical Research Council (mMRC) dyspnoea score in both cohorts, with oxygen saturation (via pulse oximetry), total lung capacity (TLC) and residual volume (RV) in ECIPSE and with FFM, QMVC, aPWV, cIMT and SPPB measures in ERICA. Univariate and multivariate logistic regression were used to determine associations with outcomes: H-AECOPD ever occurrence and all-cause mortality in both cohorts, and a cardiovascular composite of hospitalisation and cardiovascular death in ERICA. Negative binomial regression was used to assess associations with testosterone and LOS of first H-AECOPD episode and rate of exacerbations in ERICA. TT was log-transformed for all analyses. Multivariate adjustments were the same in both cohorts: models were adjusted for age, BMI, smoking status and FEV1. Subgroup analyses by GOLD stage were conducted for univariate and multivariate analyses of H-AECOPD and all-cause mortality for both cohorts. All analyses were carried out in R studio using R V.4.0.3 for ERICA. Although patient level data from ECLIPSE were not available for this analysis, we were able to access previous analyses of patient-level data conducted by study authors; these analyses had previously been presented in abstract.^27^

Analyses were repeated using free testosterone instead of TT. The empirical free testosterone (EFT) formula was used to calculate this from TT and sex hormone binding globulin (SHBG)^14 28 29^:

EFT‐low (TT<5 nM)=−6.593+19.304×TT+0.056×SHBG−0.0959×TT×SHBG EFT‐high (TT≥5 nM)=−52.65+24.4×TT−0.704×SHBG−0.0782×TT×SHBG−0.0584×TT^2^.

Where free-t estimated from the EFT formula resulted in negative values, these were taken to be 0.001 ng/dL. SHBG in serum samples were estimated by an immunochemiluminescent assay using a Roche Platform. The LLOQ is 2 nmol/L. The reference range for men is 13 to 70 nmol/L and the coefficient of variation is 4.7%.

## Results

Data from 1296 male participants in ECLIPSE and 386 male and 244 female participants in ERICA were analysed. Mean (SD) serum testosterone levels were consistent across cohorts; 459 (197) ng/dL for males (ECLIPSE) and 455 (200) ng/dL for males (ERICA) and 28 (56) ng/dL for females (ERICA), as were age and BMI (table 1). Ten per cent of males in ERICA had clinically low serum TT levels (<250 ng/dL), but the literature was less conclusive for thresholds for low testosterone in females.^30^ We are unable to calculate this in ECLIPSE as individual participant data were not available.

**Table 1:** Baseline demographics for ECLIPSE and ERICA cohorts*

Variable	ECLIPSE		ERICA			
	Males (N=1296)		Male (N=386)		Female (N=244)	
	N		N		N	
Age (years)	1296	63.9 (7.1)	386	67.9 (7.99)	244	66.5 (7.28)
BMI (kg/m^2^)	1296	26.7 (5.2)	386	27.3 (5.26)	244	27.5 (6.39)
Height (m)			386	1.73 (0.07)	244	1.59 (0.07)
Weight (kg)			386	81.4 (17.7)	244	69.4 (17.0)
FFM (kg)			369	57.4 (9.13)	239	41.7 (5.95)
Obese (BMI>30 kg/m^2^)			386	114 (30%)	244	76 (31%)
Smoking status: current	1296	449 (35%)	386	114 (30%)	244	81 (33%)
Free testosterone (ng/dL)†			386	6.39 (2.87)	244	0.31 (0.85)
Total testosterone (ng/dL)	1296	459.1 (197.1)	386	455.0 (199.9)	244	27.5 (56.4)
FEV_1_ (L)			386	1.52 (0.542)	244	1.07 (0.353)
FEV_1_ (%)			386	51.5 (16.6)	244	53.2 (15.1)
GOLD stage:						
2	1296	542 (42%)	386	217 (56%)	244	143 (59%)
3		559 (43%)		122 (32%)		87 (35%)
4		195 (15%)		47 (12%)		14 (6%)
BODE‡ index			373	2 (1, 5)	230	3 (1, 5)
MMRC‡			386	1 (1, 3)	243	2 (1, 3)
6MWD (m)			373	359 (130)	231	324 (125)
SPPB: chair stand time (s)‡			376	12.50 (10.00, 15.83)	238	12.6 (10.05, 17.08)
Gait time (s)‡			383	4.03 (3.30, 4.76)	240	4.50 (3.70, 5.70)
QMVC (kg)			374	35.5 (10.76)	237	22.7 (7.44)
aPWV (m/s)‡			358	10.05 (8.50, 11.98)	222	9.45 (8.20, 11.18)
cIMT (mm)			351	0.851 (0.189)	222	0.829 (0.204)

*Statistics summarised using mean (SD) for continuous variables and n (%) for categorical variables unless otherwise specified.

†Free testosterone calculated by the EFT formula.^14 28 29^ Where free-t estimated from the EFT formula resulted in negative values, these were taken to be 0.001 ng/dL.

‡Statistics summarised using median (IQR).

aPWV, aortic pulse wave velocity; BMI, body mass index; BODE, body mass index(B), degree of obstruction(O), dyspnoea(D) and exercise capacity(E); cIMT, carotid intima–media thickness; ECLIPSE, Evaluation of COPD Longitudinally to Identify Predictive Surrogate End-points; EFT, empirical free testosterone; ERICA, Evaluation of the Role of Inflammation in Chronic Airways; FEV_1_, forced expiratory volume in 1 s; FFM, fat free mass; GOLD, global initiative for obstructive lung disease; LOS, length of stay; mMRC, modified Medical Research Council; 6MWD, 6 min walk distance; QMVC, quadriceps maximum voluntary contraction; SPPB, short physical performance battery.

In ECLIPSE, there were modest inverse associations between testosterone with age (r=−0.06, p=0.033), this was not observed in ERICA. Testosterone had an inverse correlation with BMI in males in ECLIPSE and ERICA (r=−0.47, p<0.001 and r=−0.43, p<0.001, respectively) and with weight (ERICA: r=−0.41, p<0.001) but not height (ERICA: r=−0.05, p=0.376). In ERICA females, testosterone was not associated with age or BMI (table 2).

**Table 2:** Correlations between total testosterone and variables in ECLIPSE and ERICA

Variable	ECLIPSE (n=1296)			ERICA (n=630)					
	Males (N=1296)			Males (N=386)			Females (N=244)		
	N	Correlation*	P value	N	Correlation*	P value	N	Correlation*	P value
Age (years)	1296	−0.059	0.033	386	0.036	0.485	244	0.037	0.562
BMI (kg/m^2^)	1296	−0.466	<0.001	386	−0.429	<0.001	244	−0.071	0.266
Height (m)	–			386	−0.045	0.376	244	−0.027	0.671
Weight (kg)				386	−0.407	<0.001	244	−0.076	0.234
FFM (kg)				369	−0.291	<0.001	239	−0.090	0.167
QMVC				374	−0.140	0.007	237	0.024	0.713
aPWV				351	0.019	0.717	222	−0.120	0.075
cIMT				351	−0.013	0.804	222	−0.012	0.855
SPPB: chair stand time (s)				376	−0.007	0.890	238	0.045	0.484
Gait speed (s)				383	−0.039	0.446	240	−0.029	0.658
6MWD	1270	0.064	0.023	373	0.073	0.160	231	−0.034	0.606
FEV_1_ (l)	–			386	−0.093	0.069	244	−0.049	0.443
FEV_1_ (% predicted)	1294	−0.028	0.310	386	−0.080	0.114	244	−0.018	0.775
BODE index	1236	0.019	0.505	373	0.031	0.557	230	−0.038	0.564
MMRC Numeric score	1266	−0.057	0.044	386	−0.055	0.278	243	−0.127	0.048
RV (% predicted)	510	0.131	0.003	–					
TLC (% predicted)	510	0.212	<0.001						

*Spearmans rank correlation coefficient.

aPWV, aortic pulse wave velocity; BMI, body mass index; BODE, body mass index(B), degree of obstruction(O), dyspnoea(D) and exercise capacity(E); cIMT, carotid intima–media thickness; ECLIPSE, Evaluation of COPD Longitudinally to Identify Predictive Surrogate End-points; ERICA, Evaluation of the Role of Inflammation in Chronic Airways; FEV_1_, forced expiratory volume in 1 s; FFM, fat free mass; LOS, length of stay; mMRC, modified Medical Research Council; 6MWD, 6 min walk distance; QMVC, quadriceps maximum voluntary contraction; RV, residual volume; SPPB, short physical performance battery; TLC, total lung capacity.

### Skeletal muscle markers

Testosterone also had an inverse association with FFM (r=−0.29, p<0.001) and quadriceps strength (QMVC) (r=−0.14, p=0.007) measured in ERICA males (table 2). Age did not affect the associations between testosterone with FFM and QMVC, however, after adjustment for BMI, these inverse associations with testosterone and BMI became positive and the association of QMVC with testosterone became insignificant (online supplemental table S3). In females in the ERICA cohort, testosterone was not associated with any skeletal muscle markers: (FFM, QMVC, 6MWD or components of SPPB) (table 2). In ECLIPSE, TT was associated with 6MWD (r=0.06, p=0.023).

### Lung function

In ECLIPSE, testosterone was associated with TLC (r=0.21, p<0.001) and RV (r=0.13, p=0.003). In ECLIPSE, there were modest inverse associations between testosterone with mMRC breathlessness score (r=−0.06, p=0.044), but this was not observed in ERICA males. There was no significant association between testosterone with FEV1% predicted or BODE Index in males. In females, other than an inverse association with mMRC breathlessness score (r=−0.13, p=0.048), testosterone was not associated with any other markers of lung function (FEV1% predicted or BODE Index).

### Cardiovascular markers

In ERICA, testosterone was not associated with vascular markers of aPWV or cIMT.

### Outcomes

The proportion of subjects experiencing at least one H-AECOPD in each cohort over follow-up was similar in males (n=412 (32%) in ECLIPSE and n=153 (40%) in ERICA, (χ^2^=0.056, p=0.812)) and n=106 (43%) for females (table 3). Testosterone level was not associated with occurrence of H-AECOPD in either cohort (table 4 and online supplemental table S4) (OR: 0.76, p=0.329 in ECLIPSE and OR (95% CI): 1.06 (073 to 1.56), p=0.779 and 0.77 (0.52 to 1.12), p=0.178, in ERICA males and females, respectively).

**Table 3:** Summary statistics for outcomes in ECLIPSE and ERICA cohorts*

Outcome	ECLIPSE		ERICA			
	Male (N=1296)		Male (N=386)		Female (N=244)	
	No of subjects	No of events†	No of subjects	No of events†	No of subjects	No of events†
≥1 H-AECOPD	1296	412 (32%)	386	153 (40%)	244	106 (43%)
Cardiovascular-related hospitalisation or death	–	–	386	138 (36%)	244	78 (32%)
Respiratory-related mortality	–	–	386	44 (11%)	244	28 (11%)
All-cause mortality	1296	112 (9%)	386	85 (22%)	244	43 (18%)
Exacerbation rate (H-AECOPD events per year)	–	–	153	0.45 (0.23, 0.86)	106	0.25 (0.21, 0.60)
LOS (days)	–	–	153	2 (1, 4)	106	3 (1, 7)

*Statistics summarised using median (IQR) for continuous variables and n (%) for categorical variables.

†Unless otherwise stated.

ECLIPSE, Evaluation of COPD Longitudinally to Identify Predictive Surrogate End-points; ERICA, Evaluation of the Role of Inflammation in Chronic Airways; H-AECOPD, acute exacerbation of chronic obstructive pulmonary disorder; LOS, length of stay.

**Table 4:** Multivariate regression models showing the OR of log transformed testosterone on outcomes in the ERICA and ECLIPSE cohort

Variable	ECLIPSE		ERICA			
	Males (1296)		Males (N=386)		Females (N=244)	
	OR*	P value	OR (95% CI)*	P value	OR (95% CI)*	P value
H-AECOPD	0.758	0.329	1.056 (0.727 to 1.560)	0.779	0.769 (0.519 to 1.121)	0.178
Respiratory-related mortality	–	–	1.121 (0.631 to 2.274)	0.724	0.627 (0.334 to 1.113)	0.128
Cardiovascular-related hospitalisation or death	–	–	0.39 (0.654 to 1.381)	0.740	0.954 (0.644 to 1.395)	0.809
All-cause mortality	0.574	0.157	0.692 (0.475 to 1.019)	0.055	0.639 (0.377 to 1.039)	0.082
			**Males (N=153)†**		**Females (N=106)†**	
			**RR (95% CI)‡**	**P value**	**RR (95% CI)‡**	**P value**
LOS (days)	–	–	0.876 (0.618 to 1.176)	0.455	0.720 (0.508 to 1.018)	0.079
Exacerbation rate (H-AECOPD events per year)	–	–	1.171 (0.845 to 1.718)	0.384	0.798 (0.517 to 1.214)	0.299

*OR for total testosterone computed from logistic regression models, adjusted for BMI, age, FEV_1_ and smoking status. CIs for ECLIPSE not available.

†n=number of individuals who had an at least one hospitalised AECOPD event.

‡RR for total testosterone computed from negative binomial regression models, adjusted for BMI, age, FEV_1_ and smoking status.

BMI, body mass index; ECLIPSE, Evaluation of COPD Longitudinally to Identify Predictive Surrogate End-points; ERICA, Evaluation of the Role of Inflammation in Chronic Airways; FEV_1_, forced expiratory volume in 1 s; H-AECOPD, hospitalised acute exacerbation of chronic obstructive pulmonary disorder; LOS, length of stay; RR, risk ratios.

There were no statistically significant associations between testosterone and H-AECOPD rates for males or females in ERICA (males (1.17 (95 % CI 0.85 to 1.72)), p=0.384, females (0.80 (95 % CI 0.52 to 1.21)), p=0.299), H-AECOPD LOS, CV hospitalisation or death, respiratory-related mortality or all-cause mortality (ECLIPSE: 0.57, p=0.157, ERICA males: 0.69 (95 % CI 0.48 to 1.02), p=0.055, ERICA females: 0.64 (95 % CI 0.38 to 1.04), p=0.082) evaluated using multivariate analyses (table 4). Since testosterone has a wide normal range, we additionally compared the H-AECOPD rates between men (ERICA males) with lower compared with normal testosterone. We dichotomised TT according to <250 ng/dL (clinically low), <300 ng/dL (low) and <400 ng/dL (low-normal) and compared each to above these thresholds (normal). There remained no statistically significant associations between TT when dichotomised into clinically low, low, low normal versus normal (1.00 (95 % CI 0.54 to 2.02), p=0.991, 1.06 (95 % CI 0.63 to 1.88), p=0.825 and 1.08 (95 % CI 0.71 to 1.67), p=0.713, respectively) (see online supplemental table S5). Online supplemental figure 1 shows the scatter plot of H-AECOPD rates against TT in males in ERICA.

10.1136/bmjresp-2022-001601.supp2 Supplementary data

Multivariate subgroup analyses stratified by GOLD stages (2 and 3+4), showed testosterone was significantly associated with all-cause mortality in GOLD stage 2 male participants only in both cohorts (OR: 0.25, p=0.007 in ECLIPSE and OR (95% CI): 0.56 (0.32 to 0.95), p=0.030 in ERICA) (table 5). However, there were no significant associations between testosterone and H-AECOPD in either cohorts or in any GOLD stage (table 5). Within GOLD stage 2 ERICA males, there was a modest correlation between 6MWD and TT (r=0.14, p=0.049), but not with QMVC, BODE Index or mMRC (online supplemental table S6). Online supplemental tables S7 and S8 show summary statistics by GOLD stage for ECLIPSE and ERICA for baseline variables and outcomes, respectively.

**Table 5:** Univariable and multivariable regression models for the effect of total testosterone on outcomes in the ERICA and ECLIPSE cohorts in GOLD stage 2 male patients with COPD

Cohort	Outcome	Model adjustment	OR (95% CI)*	P value
ECLIPSE (N=542)	ACM	Unadjusted	0.236	0.003
		Adjusted†	0.248	0.007
	H-AECOPD	Unadjusted	0.665	0.314
		Adjusted†	0.610	0.255
ERICA (N=217)	ACM	Unadjusted	0.619 (0.365 to 1.065)	0.068
		Adjusted†	0.558 (0.316 to 0.948)	0.030
	H-AECOPD	Unadjusted	1.028 (0.624 to 1.868)	0.920
		Adjusted†	1.038 (0.611 to 2.007)	0.900

*95% CIs not available for ECLIPSE.

†Adjusted for age, BMI, FEV1 and smoking status.

ACM, all-cause mortality; COPD, chronic obstructive pulmonary disease; ECLIPSE, Evaluation of COPD Longitudinally to Identify Predictive Surrogate End-points; ERICA, Evaluation of the Role of Inflammation in Chronic Airways; FEV_1_, forced expiratory volume in 1 s; GOLD, Global Initiative for Obstructive Lung Disease; H-AECOPD, hospitalised acute exacerbation of chronic obstructive pulmonary disorder.

Results did not materially differ when analyses were repeated with free-testosterone values^14 28 29^ instead of TT (online supplemental table S9), in particular the association with free testosterone and all-cause mortality in GOLD stage 2 males was similar. The association of free testosterone with H-AECOPD in GOLD stage 2 males was significant when unadjusted, however, after adjusting for confounding factors this was no longer the case (online supplemental table S10).

## Discussion

In two separate large COPD cohorts with baseline measurement of testosterone and longitudinal data pertaining to health outcomes, we demonstrated that circulating testosterone levels are not associated with COPD exacerbations, cardiovascular outcomes or all-cause mortality. However, in both ECLIPSE and ERICA, male participants with moderate airflow limitation (GOLD stage 2), an inverse association between testosterone levels and all-cause mortality was observed with and without adjustment for age, BMI, current smoking and FEV1% predicted.

The independent inverse association of testosterone levels with increased risk of all-cause mortality in males with COPD with moderate airflow limitation only, in both cohorts, suggests this finding is specific to this subgroup of participants. What specific cause(s) of death may have accounted for this relationship is uncertain, given that, in the ERICA cohort, no association of testosterone with cause specific respiratory-related mortality, or cardiovascular hospitalisation or mortality was observed in GOLD stage stratified analyses, possibly due to small numbers of individuals within subgroups. Besides modest negative correlations of testosterone with FFM and 6MWD (a known predictor of mortality), there were no significant associations of testosterone with other parameters assessed (ie, quadriceps strength, SPPB, vascular markers, H-AECOPD rates or cardiovascular outcomes) in this subgroup of GOLD stage 2 males. This implies these characteristics are unlikely to be important factors in testosterone’s association with all-cause mortality in this patient group. This relationship may be consistent with the association of lower testosterone levels associated with increased all-cause mortality observed in elderly male population studies.^1 2^ However, even in general population studies, what accounts for this association of lower testosterone levels with increased mortality, is unclear—it has, however, been postulated that testosterone levels may be a non-specific marker of general health.^31^ We speculate that the lack of statistically significant association between testosterone and all-cause mortality in the male participants in the overall cohorts, and in GOLD 3 or 4 males, is because the presence of severe COPD dominates mortality in these analyses. The fact we observed no statistically significant association between testosterone and rates of H-AECOPD, or with vascular markers or cardiovascular outcomes in ERICA implies that low testosterone is unlikely to be an important pathophysiology factor or of clinical prognostic significance in these outcomes or variables. It may be noted that a retrospective non-randomised study found that testosterone replacement therapy was associated with reduced risk of respiratory-related hospitalisations in men with COPD. However, there are a number of methodological factors of this study that makes it difficult to comparable with our cohorts.^32^ Two further small studies explored the relationships between testosterone and COPD outcomes. One of these did not find any difference in the number of exacerbations between patients with low testosterone compared with a control group with normal testosterone levels. Both studies found that lower FEV1, was associated with lower testosterone levels, which has been adjusted for in our analyses however, so should account for any confounding.^33 34^

The strongest cross-sectional correlation observed was an inverse association between BMI and testosterone in males in both cohorts and was due to weight rather than height. This may be explained by increased adipose tissue with increased weight (rather than skeletal muscle), which is associated with relatively higher oestrogen than testosterone in males, this correlation has been described in other (non-disease) cohorts.^35 36^ The inverse association between both FFM and quadriceps strength with testosterone in males prior to adjustment for BMI, was an unexpected finding. Nonetheless, there are contrasting data published on this relationship of FFM with testosterone.^37 38^ Possible reasons for this may be confounding from BMI and that FFM is inclusive not only of muscle mass but connective tissue, bone and organs, and skeletal muscle has a relatively lower contribution to FFM in our cohorts of elderly men with COPD. Moreover, our population has a relatively skewed and narrow distribution of age, FFM and testosterone over which to assess these associations. Further analyses in individuals who have frequent exacerbations who experience more rapid muscle loss could test this hypothesis. Further body composition measurements such as waist and trunk circumference were not measured in this study and may have provided further insights into understanding these associations with testosterone. As for QMVC, a positive relationship between quadriceps strength and testosterone has previously been evaluated in a small cohort of males with COPD.^39^ Our study is much larger however and differences in characteristics (lower 6MWD especially) may account for the difference in findings.

These analyses are the first comprehensive assessment of circulating testosterone levels in relation to health outcomes in substantially sized COPD cohorts of males and females. The strengths of these analyses are the range of clinically important outcomes assessed and association with parameters of functional and physical capacity, muscle strength, lung function and cardiovascular risk in COPD. We elected to use continuous analyses using multivariate logistic regression models rather than splitting the cohorts in quartiles of testosterone values as has been the approach in some other population studies assessing testosterone, providing statistical power for analyses. The cohorts also reflect different COPD populations, ECLIPSE was a multinational study including sites in North America and Europe. ERICA only included sites in the UK. There are recognised limitations to this study. The main limitation being that the ECLIPSE and ERICA studies were both observational in design, and therefore, we cannot infer causality for any of the associations that we have observed in these analyses. In addition, menopause status, which may be an important factor in interpreting circulating testosterone levels for women, was not recorded. However, both studies had inclusion criterion of >40 years of age, therefore, most women eligible would have been postmenopausal. It is also likely that the number of cases of prostate cancer may be higher than recorded; in ERICA, prostate cancer was recorded in only one participant’s record on the study database, although finasteride use, a drug to treat benign prostatic hyperplasia, was recorded in 12 participants. However, given the similar results in ECLIPSE and ERICA, it seems unlikely to impact results. Patient-level data were not available from ECLIPSE to enable further in-depth statistical analysis. Different reporting methods of H-AECOPD (patient reported at follow-up for ECLIPSE, HES data for ERICA) and mortality were used, although separate analysis for individual cohorts were undertaken. Also, analyses were restricted to hospitalised exacerbations. ERICA also had a longer duration of follow-up than ECLIPSE. However, evaluating the proportion of participants who ever had an H-AECOPD for both cohorts, showed they are reasonably similar (32% for ECLIPSE, 39% for ERICA males). A higher H-AECOPD proportion in females in ERICA (43%) is consistent with other studies data regarding sex difference in COPD exacerbations.^40^ The lower baseline 6MWD and higher mMRC score in the ERICA cohort may reflect a higher morbidity cohort than ECLIPSE, despite similar FEV1% predicted. The higher mortality in ERICA is likely due to a longer period of follow-up. Despite the differences in cohort characteristics, healthcare systems and methodologies of health outcome records between the two cohorts, the consistent results observed in separate analyses of ECLIPSE and ERICA are reassuring and indicate the statistically significant of association with all-cause mortality in GOLD stage 2 is valid, as are the lack of other statistically significant associations with other health outcomes. We also undertook analyses using serum free testosterone values as well as TT presented here, and observed no significant differences in the results.

In summary, this is the first evaluation of circulating testosterone levels in both men and women with COPD and with focus on its association with clinically important health outcomes. Although levels of circulating testosterone are a biomarker of interest, given its association with muscle strength and mass and physical performance in population studies and possible protective effect in asthma, we have found in patients with COPD, it is unlikely to be a biomarker that provides significant utility in clinical assessment for patients with COPD. However, the notable association with all-cause mortality in males with COPD GOLD stage 2 in both cohorts, suggests further research on the clinical importance of circulating testosterone levels and testosterone supplementation in this select group of patients with COPD, may be useful.

## Data Availability

Data are available on reasonable request.
