# Supplementary material for: Circulating testosterone levels and health outcomes in chronic obstructive pulmonary disease: results from ECLIPSE and ERICA
Source: BMJ Open Respir Res. 2023 Jun 14;10(1):e001601. doi: 10.1136/bmjresp-2022-001601 (PMC10277522; doi:10.1136/bmjresp-2022-001601)
Supplement: Supplementary data [file bmjresp-2022-001601supp001.pdf]

Supplementary information

Table S1. ICD-10 codes to ascertain acute exacerbation in COPD in the hospital episode statistics.

ICD-10 codes	Code description	Category	Use to ascertain AECOPD usage
J22	Lower respiratory tract infection	Possible	Use if COPD diagnosed in primary care data in First position of any finished consultant episode
J41	Simple and mucopurulent chronic bronchitis	Possible	Use if COPD diagnosed in primary care data in First position of any finished consultant episode
J41.0	Simple chronic bronchitis	Possible	Use if COPD diagnosed in primary care data in First position of any finished consultant episode
J41.1	Mucopurulent chronic bronchitis	Possible	Use if COPD diagnosed in primary care data in First position of any finished consultant episode
J41.8	Mixed simple and mucopurulent chronic bronchitis	Possible	Use if COPD diagnosed in primary care data in First position of any finished consultant episode
J42	Unspecified chronic bronchitis	Possible	Use if COPD diagnosed in primary care data in First position of any finished consultant episode
J43	Emphysema	Possible	Use if COPD diagnosed in primary care data in First position of any finished consultant episode
J43.0	MacLeod's syndrome	Possible	Use if COPD diagnosed in primary care data in First position of any finished consultant episode
J43.1	Panlobular emphysema	Possible	Use if COPD diagnosed in primary care data in First position of any finished consultant episode
J43.2	Centrilobular emphysema	Possible	Use if COPD diagnosed in primary care data in First position of any finished consultant episode
J43.8	Other emphysema	Possible	Use if COPD diagnosed in primary care data in First position of any finished consultant episode
J43.9	Emphysema, unspecified	Possible	Use if COPD diagnosed in primary care data in First position

			of any finished consultant episode
J44	Other chronic obstructive pulmonary disease	Possible	Use if COPD diagnosed in primary care data in First position of any finished consultant episode
J44.0	Chronic obstructive pulmonary disease with acute lower respiratory infection	Definite	Any position of any finished consultant episode as per validation study
J44.1	Chronic obstructive pulmonary disease with acute exacerbation, unspecified	Definite	Any position of any finished consultant episode as per validation study
J44.8	Other specified chronic obstructive pulmonary disease	Possible	Any position of any finished consultant episode as per validation study
J44.9	Chronic obstructive pulmonary disease, unspecified	Possible	First position of any finished consultant episode as per validation study

ICD-10 = international disease classification tenth edition.

AECOPD = acute exacerbation of chronic obstructive pulmonary disease.

Table S2. ICD-10 codes to ascertain acute exacerbation in cardiovascular events in the hospital episode statistics and ONS.

ICD-10 codes	Code description
I70.2, I72, I73.9-I79	Diseases of the arteries, arterioles and capillaries
I20.0-I20.1, I20.8-I25	Coronary heart disease
I60, I61, I62, I63, I64, I65-I69, F01, G46.3-G46.7, G458, G459	All stroke
I11.0, I13.0, I13.2, I50	Heart failure
I71.3-171.9	Other vascular deaths
Adjudicated	Cardiac death

ICD-10 = international disease classification tenth edition.

Table S3. Associations between FFM and QMVC with total testosterone in males and females from ERICA.

		Males: (N = 386)			Females (N= 244)		
**Primary predictor**	**Adjustment**	**N**	**Coefficient**	**p-value**	**N**	**Coefficient**	**p-value**
**FFM**	**Unadjusted**	369	-0.009 (-0.016, -0.002)	0.015	239	-0.005 (-0.020, 0.011)	0.552
	**Age (years)**	369	-0.009 (-0.016, -0.002)	0.017	239	-0.004 (-0.020, 0.012)	0.619
	**Age (years) + BMI (kg/m^2)**	369	0.010 (0.000, 0.020)	0.046	239	0.010 (-0.015, 0.035)	0.440
**QMVC**	**Unadjusted**	374	-0.006 (-0.012, 0.000)	0.043	237	0.006 (-0.006, 0.018)	0.303
	**Age (years) + BMI (kg/m^2)**	374	-0.006 (-0.012, 0.000)	0.049	237	0.007 (-0.005, 0.019)	0.227
	**BMI (kg/m^2)**	374	0.000 (-0.007, 0.006)	0.964	237	0.008 (-0.004, 0.020)	0.190

FFM = fat-free mass, QMVC = quadriceps maximum voluntary contraction, BMI = body mass index

Table S4. Univariate regression for log-transformed total testosterone in ECLIPSE and ERICA.

	**ECLIPSE**		**ERICA**			
	**Males (N=1,296)**		**Males (N=386)**		**Females (N=244)**	
**Variable**	**OR**	**p-value**	**OR (95% CI)**	**p-value**	**OR (95% CI)**	**p-value**
**H-AECOPD**	0.514	0.054	1.031 (0.747, 1.447)	0.854	0.835 (0.579, 1.187)	0.322
**Respiratory related mortality**	-	-	1.604 (0.887, 3.322)	0.166	0.754 (0.416, 1.313)	0.337
**Cardiovascular related hospitalisations or death**	-	-	0.785 (0.563, 1.087)	0.145	0.968 (0.661, 1.402)	0.866
**All-cause mortality**	0.993	0.979	0.853 (0.601, 1.244)	0.385	0.780 (0.475, 1.240)	0.309
			**Males (N=153)***		**Females (N=106)***	
			**RR (95% CI)**	**p-value**	**RR (95% CI)**	**p-value**
**LOS (days)**	-	-	0.928 (0.667, 1.232)	0.662	0.623 (0.480, 0.945)	0.044
**Exacerbation rate (H-AECOPD events per year)**	-	-	1.242 (0.893, 1.798)	0.228	0.849 (0.564, 1.267)	0.427

*N=number of individuals who had an AECOPD event

LOS = length of stay, H-AECOPD = acute exacerbation of COPD (coronary obstructive pulmonary disorder).

Table S5. Three fully adjusted multivariate negative binomial models to assess the difference in H-AECOPD between dichotomised total testosterone.

Dichotomised total testosterone threshold	Coefficient (95% CI)*	p-value
**<250 ng/dL vs ≥250 ng/dL**	0.996 (0.542, 2.023)	0.991
**<300 ng/dL vs ≥300 ng/dL**	1.063 (0.634, 1.878)	0.825
**<400 ng/dL vs ≥400 ng/dL**	1.084 (0.709, 1.673)	0.713

*Coefficient (risk ratio) and 95% CI from negative binomial models adjusted for BMI, age, FEV_1 and smoking status.

Table S6. Correlations between total testosterone and variables in GOLD stage II males in ERICA.

	ERICA Males: Gold Stage II (n = 217)		
**Variable**	**N**	**Correlation***	**p-value**
**Age (years)**	217	0.093	0.175
**BMI (kg/m²)**	217	**-0.388**	**<0.001**
**Height (m)**	217	-0.014	0.834
**Weight (kg)**	217	**-0.372**	**<0.001**
**FFM**	211	**-0.272**	**<0.001**
**QMVC**	211	-0.063	0.364
**aPWV**	207	0.019	0.781
**cIMT**	202	-0.075	0.288
**SPPB: Chair stand time (s)**	210	-0.019	0.787
**Gait speed (s)**	214	-0.103	0.132
**6MWD**	211	**0.135**	**0.049**
**FEV$_1$ (l)**	217	-0.105	0.125
**FEV$_1$ (%)**	217	-0.083	0.224
**BODE index**	211	0.013	0.852
**mMRC Numeric score**	217	-0.108	0.112

*Spearmans rank correlation coefficient.
BMI = body mass index, FFM = fat-free mass, FEV$_1$ = forced expiratory volume (in one minute), BODE = body-mass index [B], degree of obstruction [O], dyspnea [D], and exercise capacity [E], mMRC = modified medical research council, 6MWD = six minute walk distance, SPPB = short physical performance battery, QMVC = quadriceps maximum voluntary contraction, aPWV = aortic pulse wave velocity, cIMT = carotid intima-media thickness, LOS = length of stay.

Table S7. Baseline demographics for males in ERICA by GOLD stage*.

	ERICA (Male N=386)					
	GOLD Stage 2 (N=217)		GOLD Stage 3 (N=122)		GOLD Stage 4 (N=47)	
**Variable**	**N**		**N**			
**Age (years)**	217	68.2 (8.04)	122	68.3 (8.20)	47	65.9 (6.97)
**BMI (kg/m^2)**	217	28.4 (4.87)	122	26.3 (5.46)	47	24.4 (4.93)
**Height (m)**	217	1.73 (0.07)	122	1.72 (0.07)	47	1.72 (0.06)
**Weight (kg)**	217	85.1 (16.5)	122	78.3 (18.0)	47	72.2 (17.7)
**FFM (kg)**	211	59.4 (8.08)	115	55.6 (9.72)	43	53.0 (9.99)
**Obese**	217	76 (35%)	122	31 (25%)	47	7 (15%)
**Smoking status: Current**	217	56 (26%)	122	49 (40%)	47	9 (19%)
**Free Testosterone (ng/dL)**	217	6.39 (2.63)	122	6.39 (3.00)	47	6.34 (3.53)
**Total Testosterone (ng/dL)**	217	440 (177)	122	468 (220)	47	494 (239)
**FEV_1 (L)**	217	1.88 (0.38)	122	1.19 (0.24)	47	0.70 (0.15)
**FEV_1 (%)**	217	63.7 (9.02)	122	40.6 (5.69)	47	23.5 (4.45)
**BODE index†**	211	1 (0, 2)	118	4 (3, 5)	44	7 (5, 8)
**mMRC Numeric score†**	217	1 (1, 2)	122	2 (1, 3)	47	3 (2, 3)

**6MWD (m)**	211	391 (126)	118	335 (126)	44	266 (101)
**SPPB: Chair stand time (s)** †	210	12.17 (10.00, 15.34)	119	12.56 (10.10, 16.30)	47	13.20 (10.60, 18.11)
**Gait time (s)** †	214	3.83 (3.21, 4.50)	122	4.13 (3.50, 5.10)	47	4.60 (3.85, 5.50)
**QMVC (kgf)**	211	38.2 (11.0)	117	32.9 (9.55)	46	30.1 (8.91)
**aPWV (m/s)** †	207	9.8 (8.5, 12.1)	111	10.0 (8.3, 11.8)	40	11.2 (8.9, 12.2)
**cIMT (mm)**	202	0.86 (0.19)	108	0.85 (0.21)	41	0.81 (0.16)

*Statistics summarised using mean (SD) for continuous variables and n (%) for categorical variables unless otherwise specified.
†Statistics summarised using median (IQR)
BMI = body mass index, FFM = fat-free mass, FEV_1 = forced expiratory volume (in one minute), BODE = body-mass index [B], degree of obstruction [O], dyspnea [D], and exercise capacity [E], mMRC = modified medical research council, 6MWD = six minute walk distance, SPPB = short physical performance battery, QMVC = quadriceps maximum voluntary contraction, aPWV = aortic pulse wave velocity, cIMT = carotid intima-media thickness.

Table S8. Summary statistics of outcomes by GOLD stage in males from ECLIPSE and ERICA*.

			GOLD stage		
**Cohort**	**Sex**		**2**	**3**	**4**
**ECLIPSE**	**Males**	**N**	542	559	195
		**H-AECOPD**	30 (5.5%)	54 (9.7%)	28 (14.4%)
		**All-cause mortality**	97 (18%)	210 (38%)	105 (54%)
**ERICA**	**Males**	**N**	217	122	47
		**H-AECOPD**	52 (24%)	65 (53%)	36 (77%)
		**Respiratory related mortality**	8 (4%)	16 (13%)	20 (43%)
		**Cardiovascular related hospitalisation or death**	71 (33%)	47 (39%)	20 (43%)
		**All-cause mortality**	31 (14%)	31 (25%)	23 (49%)
		**N**	52	65	36
		**Exacerbation rate (H-AECOPD events per year)**	0.55 (0.77)	0.58 (0.43)	0.94 (0.73)
		**LOS (days)**	2.71 (4.93)	4.68 (7.77)	4.31 (6.66)

*Statistics summarised using mean (SD) for continuous variables and n (%) for outcome variables.
H-AECOPD = acute exacerbation of coronary obstructive pulmonary disorder, LOS = length of stay.

Table S9. Multivariate regression models showing the odds ratio of log transformed free testosterone on outcomes in the ERICA and ECLIPSE cohort.

	**ERICA**			
	**Males (N=386)**		**Females (N=244)**	
**Variable**	**OR (95% CI)***	**p-value**	**OR (95% CI)***	**p-value**
**H-AECOPD**	0.970 (0.893, 1.053)	0.469	0.637 (0.217, 1.053)	0.250
**Respiratory related mortality**	0.995 (0.878, 1.125)	0.933	0.113 (0.006, 0.913)	0.107
**Cardiovascular related hospitalisation or death**	1.000 (0.921, 1.087)	0.993	1.128 (0.817, 1.660)	0.450
**All-cause mortality**	0.907 (0.824, 0.996)	0.043	0.221 (0.025, 0.915)	0.143
	**Males (N=153)†**		**Females (N=106)†**	
	**RR (95% CI)‡**	**p-value**	**RR (95% CI)‡**	**p-value**
**LOS (days)**	0.993 (0.926, 1.066)	0.842	0.354 (0.127, 1.031)	0.054
**Exacerbation rate (H-AECOPD events per year)**	0.990 (0.927, 1.057)	0.768	0.980 (0.285, 2.472)	0.971

*Odds ratios (OR) for free testosterone computed from logistic regression models, adjusted for BMI, age, FEV_1 and smoking status. Information on free testosterone was not available for ECLIPSE.

†N=number of individuals who had an at least one hospitalized AECOPD event

‡Risk ratios (RR) for total testosterone computed from negative binomial regression models, adjusted for BMI, age, FEV_1 and smoking status.

H-AECOPD = hospitalized acute exacerbation of chronic obstructive pulmonary disorder, LOS = length of stay, FEV_1 = forced expiratory volume (in one second).

Table S10. Univariable and multivariable regression models for the effect of free testosterone on outcomes in the ERICA cohort in GOLD Stage 2 male COPD patients.

Cohort	Outcome	Model adjustment	Odds Ratio (95% CI)	p-value
**ERICA (N=217)**	**ACM**	Unadjusted	0.846 (0.723, 0.983)	0.032
		Adjusted*	0.827 (0.703, 0.965)	0.018
	**H-AECOPD**	Unadjusted	0.874 (0.770, 0.988)	0.034
		Adjusted*	0.879 (0.769, 1.000)	0.054

*Adjusted for age, BMI, FEV_1 and smoking status
ACM = all-cause mortality, H-AECOPD = hospitalized acute exacerbation of chronic obstructive pulmonary disorder, FEV_1 = forced expiratory volume (in one second).
