# Supplementary figures and images for: Circulating testosterone levels and health outcomes in chronic obstructive pulmonary disease: results from ECLIPSE and ERICA
Source: BMJ Open Respir Res. 2023 Jun 14;10(1):e001601. doi: 10.1136/bmjresp-2022-001601 (PMC10277522; doi:10.1136/bmjresp-2022-001601)

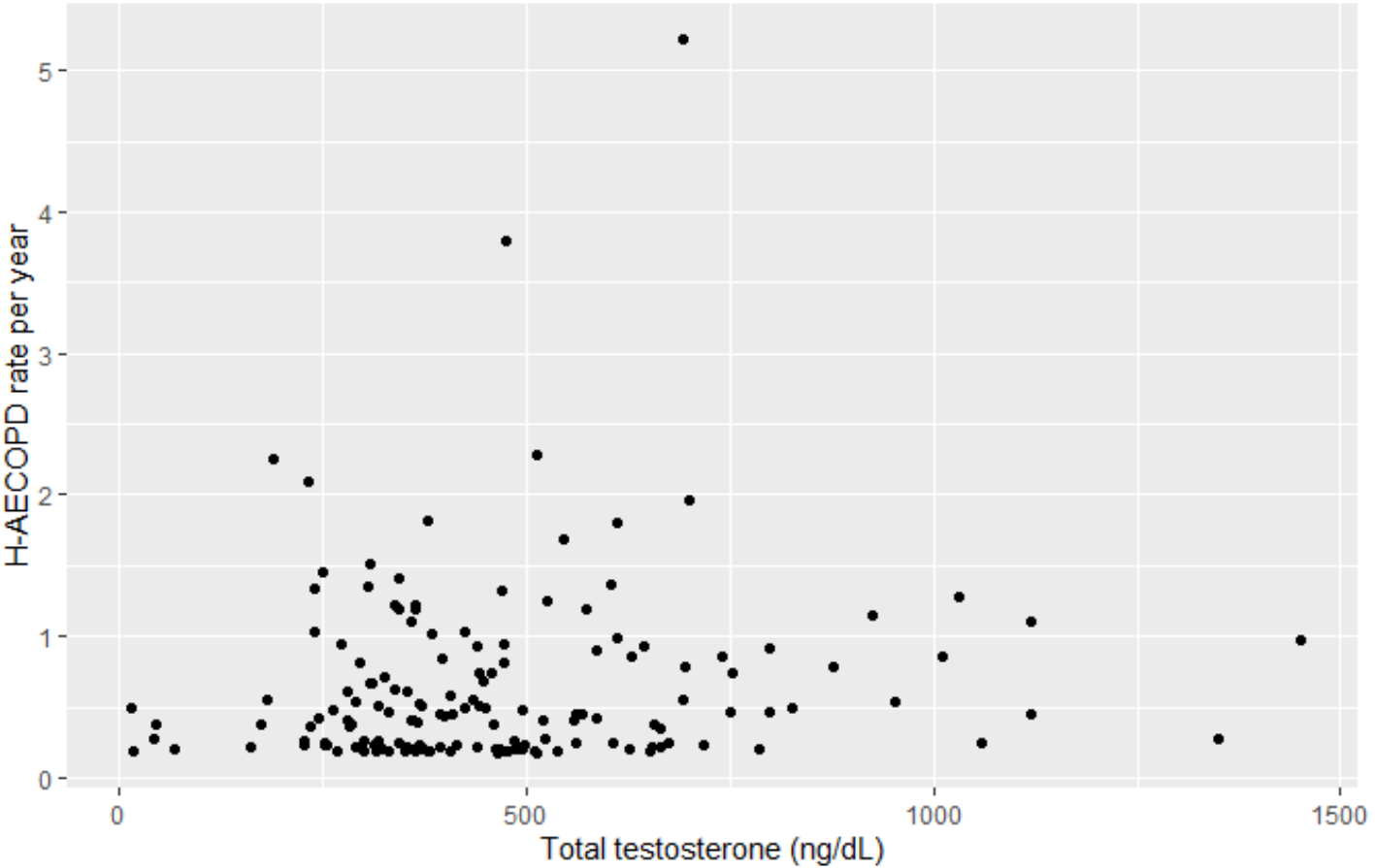

Supplement: Supplementary data [file bmjresp-2022-001601supp002.pdf]
